# Structural Dimension Exploration and Measurement Scale Development of Employee Involution in China’s Workplace Field

**DOI:** 10.3390/ijerph192114454

**Published:** 2022-11-04

**Authors:** Guoqin Dou, Guangxia Li, Yunyun Yuan, Bin Liu, Lifeng Yang

**Affiliations:** 1 School of Information Engineering, Fuyang Normal University, Fuyang 236037, China; 2 School of Economics, Fuyang Normal University, Fuyang 236037, China; 3 School of Management and Economics, Beijing Institute of Technology, Beijing 100000, China; 4 School of Business, Fuyang Normal University, Fuyang 236037, China

**Keywords:** Chinese employees, involution, grounded theory, factor analysis, scale development

## Abstract

The phenomenon of workplace involution has attracted ample attention. How to make employees treat their work with the correct attitude and behavior and improve their work performance has become a realistic proposition. This study uses a combination of qualitative and quantitative research methods, with the help of grounded theory, to conduct an exploratory study on the structural dimensions of employee involution in the Chinese workplace and, on this basis, to develop and test the measurement scale. The research results show that employee involution is a multi-dimensional construct with rich connotations, including four dimensions: inefficient busyness, exhaustion of innovation, promotion anxiety, and internal competition. The measurement scale consists of four factors and 13 items. The factor analysis results showed that the developed scale’s reliability and validity reached an ideal level. To a certain extent, this study promotes the recognition and attention of various types of organizations at all levels to involution. The research conclusions provide theoretical guidance for employees to get rid of the involution crisis and will motivate managers to formulate better intervention measures to prevent and reduce workplace involution.

## 1. Introduction

It is not your boss who makes you work overtime, but other people willing to work overtime [1,2]. This sentence pointedly pointed out that employees working overtime have evolved into a group self-iteration that is independent of the boss’s requirements, which will eventually force employees to enter the “prisoner’s dilemma” in the workplace [3]. In the modern workplace, fierce internal competition, repetitive single-work content, disproportionate income returns, and the two-way effects of employee self-pressure and self-exploitation make the dilemma of inefficient busywork and lack of innovation more and more common. Affected by the COVID-19 epidemic, the world economy, society, culture, education, etc., are obviously shrinking inward, domestic and international environmental pressure is increasing, and many industries are in a development dilemma stage [4,5]. With the increase of employees’ internal pressure, the concentration of the competitive field, and the decrease of self-identity [6], the increasing external competitive pressure of the company is constantly transformed into employees’ internal self-consumption, making the involution problem particularly prominent in the workplace. At the moment, promoting personal health and well-being should be not only a social and political goal but also a key management goal [7,8]. Therefore, how to prevent employees from generating negative involution emotions and instead maintain enthusiasm for work and correct work behaviors has become a management proposition of great practical significance. How to formulate reasonable organizational policies and operational involution interventions to help employees get rid of the vortex of involution has also become a topic that needs to be discussed urgently. This premise is to explore the connotation and structure of employee involution and identify the essence of involution in theory. However, there is still a lack of research on the conceptualization and theoretical discourse of “involution” in academic circles, and the structural analysis and conceptual essence of “involution” have not yet been formed. In view of this, this article conducts a systematic study on employee involution, and clearly defines its connotation and measurement methods, to promote the research progress of involution in the workplace.

As a verb, “involution” becomes more and more inward, and the living space becomes narrower and narrower [9]. Originally used to describe shells that twist inward as they grow, forming a complex structure [10]. This vividly depicts the living conditions of people under fierce internal competition, and it also increasingly arouses the resonance and recognition of employees under the “996” and “007” working systems. In recent years, Chinese companies and organizations have normalized overtime culture, such as “advocating struggle” and “advocating labor” [11,12]. However, time maximization does not mean the maximization of production capacity. For an enterprise, involution is not development but duplication and internal consumption without creation [13]. Prior studies have shown that long-time work will increase employees’ fatigue and aggravate negative emotions such as stress and anxiety [14,15], and it will also reduce employee well-being [16]. Individuals with anxiety and stress tend to interpret the stimuli and events in their environment as excessive threats [17,18]. Extensive empirical evidence shows that work-related stressors contribute to the onset or deterioration of depression [19,20] and stress-related diseases [21,22]. It can be seen that the appearance of involution is that employees seem to be full of enthusiasm and busyness, but it is actually a serious attrition of work and enterprise enthusiasm, which is not conducive to the healthy development of employees.

As far as individual employees are concerned, when they enter the workplace full of passion for work and are eager to achieve career success, they are overwhelmed by each other and have internal friction when faced with strong social pressure and intense competition [5]. Some voluntarily opted for involution in ways that exceeded actual needs, and some were forced to join it. Under the limited cognition and resources, in order to win the peer competition and strive for high-quality resources, the employees continuously refine and complicate the input of unit resources, gradually forming the diminishing marginal benefits under irrational competition, resulting in the decline of individual “return on pay”. According to the effort-reward imbalance model of Siegrist [23], if an employee’s efforts at work are not reciprocated, they may feel dissatisfied at work, which can manifest as a loss of happiness. When the workplace struggle evolves into excessive overtime and intensive work, employees lose their valuable goals, future life plans, and value pursuits in the process. However, when their struggle was questioned and denied, they were deeply frustrated and gradually changed to “Buddha” or “lying flat” to adjust their state, and the willpower of young people also tended to dissipate.

As far as business managers are concerned, rather than lying flat, they prefer that employees have a certain degree of involution. Under the system’s constraints, employees, managers, and even the entire enterprise tend to make “rational” choices and participate in an internal competition in order to maximize their interests. Internal competition can produce constructive organizational outcomes [24,25] and achieve higher levels of innovation and performance across the organization [26]. Short-term involution may be helpful for organizational development, but in the long run, employees’ work motivation dominated by involution will become more and more utilitarian, which will lead to irrational internal competition and employees’ mental state of being tired of coping. The negative impact of this psychological state is particularly serious for high-risk work such as mining, blasting, and quarrying [27,28]. When faced with a working environment with tight deadlines, high requirements, and security threats, the employees who are dominated by involution lack unity, cohesion, and positive emotions, or they are eager to make quick profits or are tired of coping, ignoring the original professional skills and psychological quality. It is likely to cause the personal safety and health of employees to be damaged eventually. Therefore, when an organization ignores the complexity and continuity of employees’ mental and physical states, involution manifests in overwhelming anxiety and mental fatigue in the will rather than continuous excitement and vitality in the fierce competition [29].

Everyone is talking about involution, but there is still no clear definition of “involution”. So, what is employee involution? What is included? What are the deep-seated factors that induce employee involution? How to evaluate the differences in the degree of involution among different groups? Answering these practical issues requires an in-depth discussion of the connotation, structural dimensions, and measurement of employee involution and has fundamental significance for the research and development of involution. Based on the above background, this study believes that it is necessary to deeply explore the internal and external factors that affect the formation of employee involution and the mechanism of their action on individual behavior motivation from a micro perspective and then formulate targeted and effective guidance and intervention policies. Therefore, the research on the connotation and structure of employee involution in the workplace has excellent policy guiding significance for enterprises to guide employees to overcome involution more effectively.

## 2. Literature Review

In 2020, the term “involution” became popular on the Internet. People have a relatively common emotional resonance with “involution” from campus to society, from students to corporate employees. They feel confused about the future due to the competitive pressure brought about by “constantly beating their own gyroscopic endless loop”. The popularity of “involution” is a true portrayal of the enormous pressure faced by youth groups and major small and medium-sized enterprises in the process of job hunting and survival under the influence of the epidemic. So what is involution? Involution, also known as “over-densification”, has the verb form involveere, which means “wrapping and twisting”. This concept was first used to emphasize the process of continuous refinement and complication in the original state due to the inability to achieve steady state and mode conversion for a certain thing under the constraints of external conditions [30]. Subsequently, the concept of “agricultural involution” was further developed to explain the failure of industrialization in Java [31]. In China, some scholars associate involution with the diminishing marginal returns of labor, opening analysis of “involution” in Chinese society [32]. Subsequently, other scholars proposed “involution of state power” [33], and involution was extended to different fields and became one of the high-frequency concepts in sociological research. The definition of “Involution” in western academic circles is shown in Table 1.

Through the above-mentioned literature, it is found that American scholars first proposed the concept of involution, which was later evolved by other scholars, and then transformed by domestic scholars, making it quite controversial [34]. However, the “involution” of “everyone can be involved, and everything can be involved” in the current Chinese popular context has deviated from the original academic meaning to a certain extent. Social platforms and we-media have highly simplified the rich connotation of the word “involution” and referred to the white heat of competition. In fact, “involution” in the academic context basically includes two elements: one is the concept of limited external expansion, and the other is the reduction of internal marginal benefits. In recent years, some media have also reported the interpretation of involution in Western contexts. For example, Liu [35] described the state of involution “985” college students as “anxious, stressed, overworked, trapped in a status race”. Fan Wang and Yitsing Wang [36] interpreted it loosely as “a feeling of burn out”. The most official explanation is Qin Gang [37], the Chinese ambassador to the United States, who interprets “involution” as “irrational internal competition, or competition ‘voluntarily’, where individuals struggle to suffer inflation. It can be seen that “involution”, as an academic concept with interdisciplinary influence, was first applied by scholars in anthropology, economics, sociology, political science, and other multi-disciplinary fields, and has become an imperative theoretical paradigm, analytical perspective, and analytical tool. It has been used by foreign scholars in various “non-evolutionary” urban and rural social studies. However, in today’s China, the interpretation of China’s involution is more inclined to the individuals’ psychological and behavioral deviations caused by irrational competition. Under the involution, there is no real winner, and everyone is in a dead cycle of energy consumption.

From the perspective of the Chinese workplace, “involution” is no longer a negative phenomenon reflected in work but has gradually become a social emotion that is growing and spreading. The “involution” mentality is especially prevalent in some highly competitive, time-critical jobs. Under the double pressure of the external environment (work environment and work system, etc., employees cannot get rid of the current development dilemma, innovate but not innovate at work, and cannot provide a friendly environment for their development) and internal environment (the development and progress of employees in work meet a bottleneck, they are physically and mentally exhausted in repeated self-consumption, and the competition pressure is large and lack of motivation), they are prone to stress, anxiety, and depression [38], which stimulate employees to produce job-deflecting behaviors [39], and undermining employees’ creativity [40]. Research on work stress has confirmed that employees who have been under work stress for a long time are more likely to be dissatisfied with their jobs and that job performance negatively correlates with it [41,42]. In addition, employee involution is closely related to job burnout, which is a state of exhaustion produced by individuals who doubt their professional value and their work ability [43]. Job burnout, which consists of emotional exhaustion, low achievement, and dehumanization, negatively predicts cognitive and emotional well-being [44]. However, there is a certain difference between involution and slackness, discouragement and bleakness emanating from “stagnation”, “decline”, and “depression”. Involution emphasizes long-term continuity and diversity and is a “stagnation” in a continuous and aggressive state. Employees trapped by involution strive for high-quality resources through extreme exploitation of themselves and shrewd calculation of the rules. The competition among organizational members gradually evolves into a “quantity” competition between work hours and content. They consume too much intelligence, energy and physical strength but do not gain substantial benefits [13]. Instead, it arouses employees’ negative attitudes towards work and the work environment [45] and negative beliefs about work performance (e.g., a sense of failure in work, lack of value, and low self-efficacy; [6]). Therefore, employee involution may cause a range of negative responses, ranging from emotional dissatisfaction and depression to reduced morale and work performance, which not only causes internal injury to individuals but also is not conducive to the organization’s long-term development.

Although the concept of “involution” has been widely used by domestic and foreign scholars in various fields, the research on “involution” in the current literature is relatively scattered, and there has never been a clear definition of “involution”. The research on its dimension is even less, and no systematic research has been conducted, which seriously hinders the development of involution. In view of this, this study is formally based on the needs of the practice and theoretical gaps, with case studies as the core, to systematically refine the structural dimensions of involution and, on this basis, develop an effective measurement scale with a view to promoting the research progress of involution in the workplace.

## 3. Study Design and Data Sources

### 3.1. Research Methods: Grounded Theory

Grounded theory [46] is a research method that analyzes empirical data and constructs theories from the bottom up through a process of heuristic abstraction [47] that contributes to the formation of substantive or formal theories. The aim is to generate theories by systematically collecting and analyzing data rather than testing preconceived ideas or hypotheses [48,49]. Despite the qualitative research approach, it combines the depth and rich interpretation of qualitative research with the logic and rigorous systematic analysis of quantitative research [47,50]. According to the practical version of the grounded theory, the analysis link is mainly divided into open coding, axial coding, and selective coding. In the process of data analysis, the idea of constant comparison analysis is adopted to continuously compare data with data and theories with each other until a new substantive theory is developed.

The reasons why this paper chooses grounded theory as the research method are as follows: first, most of the existing involution research is quantitative and lacks qualitative exploratory analysis. Second, various factors can affect employee involution, so traditional hypothesis testing is difficult to conduct. Finally, the relationship between the causes of involution and its influencing factors is complex, so it is difficult for quantitative studies to clearly explain the “how” and “why” relationships between them. Because the rooting theory is explanatory in nature, aiming to discover concepts and relationships and provide theoretical explanations for existing phenomena [48], this study believes that rooted in rich data and specific research situations, the concepts in the data can be deeply summarized and abstracted from bottom to top, and the structural dimension of employee involution can be extracted.

### 3.2. Data Collection and Processing

Since there are few empirical studies on the influencing factors of employee involution and no well-established measurement scales, it is not possible to collect data using structured scales. Therefore, this study obtained first-hand data by designing unstructured questionnaires (open-ended questionnaires) to conduct in-depth interviews with representative employees in different industries, that is, to learn employees’ cognition and attitude towards involution. The method of theoretical sampling is used to select representative employees who have a certain understanding and opinion of the involution and have a certain level of educational knowledge. At the same time, it pays attention to the reasonable distribution of the gender, age, occupation, and other structures of the interviewees, which is in line with reality. The determination of the sample size is based on the principle of theoretical saturation. That is, samples are drawn until the newly drawn samples no longer provide new important information.

From the existing qualitative research literature, scholars mostly use face-to-face interviews to obtain the required information. In this study, in addition to the usual face-to-face interviews, the QQ platform and WeChat platform are also used for interviews. This kind of online interview has the following advantages. It is not necessary for the interviewer to meet the interviewee directly, and its implementation is more convenient and free from time and space constraints. At the same time, the interviewees will not feel constrained, and their answers will be more free and truthful. They will not be easily affected by the interviewees’ oral language and behavioral language. Moreover, the answers are often thoughtful and logical. Therefore, this study adopts a combination of one-to-one, face-to-face interviews, QQ platform, and WeChat platform online interviews to conduct individual in-depth interviews. In the in-depth interview, this study uses the problem-focused interview method. That is, the interviewer guides the interviewee to focus on the interview topic from their own perspective by establishing a participatory dialogue method during the interview. The time for in-depth interviews is set at about half an hour, and relatively sufficient room for thinking and expression is reserved for the interviewees. Before the formal interview, the interviewers first explain the topics and precautions of the interview to the interviewees and conduct communication and discussion. The interview is based on the principles of openness, interaction, and confidentiality. According to the requirements of grounded theory, there is no presupposition and paradigm in the interview, but a simple interview outline is set in advance to improve the efficiency of the interview. The in-depth interview mainly focuses on the following issues:

1. Have you ever learned about involution in the current popular context? How do you view the phenomenon of involution or talk about your understanding of the term involution?
2. What do you think are the main manifestations of employee involution in the workplace? Can you talk about your own behavior or that of your colleagues?
3. In your opinion, what are the factors that cause employee involution in the workplace? How do these factors affect employee involution?
4. Do you think you are currently in a state of involution? Can you describe it in detail in relation to your daily life?
5. If you are in the involution state, what changes do you think involution has brought to you in psychology and behavior? How did you cope?

During the interview, further follow-up questions will be asked around the above questions and the captured conceptual categories so as to deeply understand the internal psychology of the interviewees and further extend the interview content. According to the principle of theoretical saturation, a total of 103 interviewees were selected. The basic information of the interviewees is shown in Table 2.

## 4. Data Analysis

### 4.1. Open Coding

In the process of grounded coding, in order to ensure the reliability and validity of the research, the operation is strictly carried out according to the grounded coding technical procedure of Strauss and Corbin [48]. Open coding is the process of analyzing the collected data, analyzing the true connotation of the original data, conceptualizing and refining the complicated original data, and forming a category to achieve data aggregation. After sorting out the interview records, 2/3 of the interview records were randomly selected for grounded coding analysis, and the other 1/3 of the interview records were tested for grounded theory saturation. The study invited five researchers from related majors to sort out the interview data and collected a total of 1652 expressions of “involution”. In order to deeply explore the structural dimension of the involution, this paper excludes the relatively simple and too vague answer sentences in the interview records and finally obtains more than 900 original sentences and corresponding initial concepts. Due to a large number of initial concepts and the overlap to a certain extent, this paper selects initial concepts with a repetition frequency of more than five times for categorization and eliminates individual inconsistent initial concepts. Open coding started operation at the end of the first interview, in the first interview to sort out some concepts first, then analysis of the correlation and differences between these concepts, and then concludes some category, and then according to the problems found during the encoding process and sort out the concept of category, targeted to begin a second interview. This is done regularly until the coder feels that the concepts and categories of coding are relatively rich and relevant concepts and categories constantly appear in the coding process. Then the interview can be stopped, and the coding can proceed to the next level. This process also includes manual coding comparison and analysis at the same time and finally forms an open coding table. The excerpts are shown in Table 3.

### 4.2. Axial Coding

Axial coding is to discover and establish various relationships between various conceptual categories. In the specific analysis process, firstly, the 13 categories formed in the open coding stage are further clustered and summarized to determine whether there are potential connections in the meaning and logical relationship of each category. Then, on this basis, the subordination between categories is determined, the main category and the sub-category are developed, and the logical subordination structure between categories is established. Through the above links, four main categories are determined in this stage. The axial coding results are shown in Table 4.

### 4.3. Selective Coding

Selective coding refers to excavating the core category from the main category and systematically establishing the connection relationship between the core category and other categories. In the process of continuous comparative analysis, the core category must be repeatedly proved that it is dominant to most categories, can clearly describe the relationship between most categories, and can include most categories within a comprehensive theoretical framework. Through in-depth exploration, the typical relationship structure between the main categories formed after selective encoding is shown in Table 5 below.

### 4.4. Saturation Test

Theoretical saturation test refers to the further development of a certain category feature as an identification criterion for stopping sampling without obtaining additional data [51]. The theoretical saturation test of this paper is carried out with 1/3 of the interview records reserved. After coding analysis, no new important categories were found, and no new constituent factors were found within the four main categories, and the research results reached theoretical saturation.

## 5. Definition of Involution Concept Structure

Based on the analysis of the case materials and the grounded theory, this study considers that employee involution is the phenomenon that the employees, with limited cognition and resources, continue to fall into the working state of simple mechanical repetition and are tired of coping due to the lack of work ideas, innovation consciousness, and direction of struggle, resulting in low work efficiency and long-term stagnation. Therefore, we define employee involution as the state of “voluntary” competition for work in the workplace, in which employees face internal competitive pressure and are tired of working tasks, constantly falling into energy and physical energy consumption, and are accompanied by doubts about self-efficacy and ability improvement.

**Figure 1:**
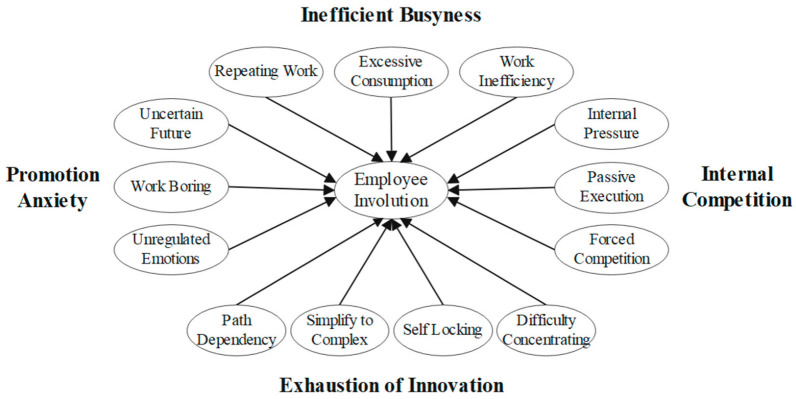
Involution Impact Factor Model.

Employee involution includes four dimensions: inefficient busyness, exhaustion of innovation, promotion anxiety, and internal competition. (1) Inefficient busyness is the behavioral manifestation of employees’ involution, including repeating work, excessive consumption, and work inefficiency. The specific manifestation is that employees are in a state of excessive work consumption and stay in a state of inefficient self-repetition for a long time; (2) Exhaustion of innovation is the cognitive performance of employees’ involution, including four aspects: path dependence, simplify to complex, self-locking and difficulty concentrating. Due to the limitations of cognition, when employees are faced with complex decisions, their thinking patterns will rely on past scenarios to make explanations and reactions. When employees cannot expand or change themselves, they will only develop in the direction of internal complexity. Even though the work is very complicated, it is always copying itself, lacking the desire and motivation for innovation; (3) Promotion anxiety is the emotional manifestation of employees, which includes three aspects: uncertain future, boring work, and unregulated emotions. Boring, repetitive work, difficulty in improving work efficiency, and low level of competition make employees’ negative emotions increasingly prominent; (4) Internal competition is the performance of employees’ involution in motivation, including internal pressure, passive execution, and forced competition. In most organizations today, the dense internal environment forces employees to participate in increasingly intense competition and experience continuous pressure but cannot quit. The theoretical model of the involution influence factor is shown in Figure 1.

## 6. Development and Testing of Involution Measurement Scales

### 6.1. Compilation of Scales

#### 6.1.1. Compilation of Initial Items

During the compilation of the initial items, based on the connotation of the four dimensions mentioned above, sentences with similar meaning expressions to the basic connotation of each category are selected from the original materials for processing and sorting. On this basis, a total of 42 initial items are formed, and the measurement items of each dimension are guaranteed to be more than three.

#### 6.1.2. Consolidation and Simplification of Items

The initial items formed in the above stages still have a certain degree of repetition in sentence description and meaning expression. Therefore, 42 initial items were combined and deleted in this study. The specific operation process is as follows: first, five doctoral students and postgraduate students majoring in management were invited to merge and delete 42 items in a “back-to-back” way from the two aspects of “whether semantic repetition” and “whether it is related to a construct”. In this link, the items that were agreed upon were deleted first, and the inconsistent and uncertain items were discussed and agreed upon. Based on this, 21 items were retained. Then, on this basis, two professors in the field of organization and human resources research were invited to review and revise the items and delete items that are not specific enough in expression, or that are vague in meaning and weakly related to the theme, to ensure the accuracy of the measurement of the scale. Finally, the scale was compiled into a questionnaire using the Likert 5-point scoring method, which was distributed to the respondents and other employees who had participated in the interviews for pre-testing, and the semantics of the questionnaire were revised and adjusted according to their feedback. Finally, an initial measurement scale with four dimensions and 15 items was formed.

### 6.2. Measurement of Scale

#### 6.2.1. Exploratory Factor Analysis

This study uses exploratory factor analysis to explore the internal structure of employee involution. The data used for statistical analysis mainly comes from employees of 20 small and medium-sized enterprises in various industries such as manufacturing, construction, service, and education in Anhui Province. Firstly, we identified the contact person of each company, explained the purpose, content, and process of the survey, and emphasized the anonymity of the questionnaire to gain the trust of the contact person. Secondly, with the support of human resource managers of various companies, we sent a letter of explanation to each participant, which mentioned the information about employee involution and the purpose of our research. Finally, in order to let employees complete the questionnaire carefully, we entrusted the human resources department to assist in ensuring the recovery rate and accuracy of the questionnaire survey.

A total of 587 questionnaires were distributed this time, and 549 were recovered. After sorting and analyzing the data of the questionnaires, after eliminating incomplete or inconsistent questionnaires, a total of 440 valid questionnaires were obtained, and the effective recovery rate was 74.95%. Among the valid samples, 46.6% were male, 71.1% were in the 40-year-old age group, and 33.5% had a bachelor’s degree or above. The demographic data of the respondents are shown in Table 6.

In order to conduct exploratory factor analysis and confirmatory factor analysis on the data, 440 valid sample data were randomly divided into two parts, 220 samples were used for exploratory factor analysis, and 220 samples were used for confirmatory factor analysis. The difference test shows that there is no significant difference in the distribution of gender, age, education, and position between the two samples. Firstly, the KMO value of 220 data was calculated, and the Barlett sphericity test was performed. The KMO value was 0.953 (>0.8), and the Barlett sphericity test was significant (Sig = 0.000), which could be used for factor analysis. In EFA, the criteria of principal component extraction factor, maximum variance method rotation, eigenvalue greater than 1, and factor load no less than 0.5 were used to gradually delete the items that did not meet the standards. Finally, 13 items were left. Four of the factors were factored out. The loading of the above 13 items ranged from 0.662 to 0.817, explaining 87.425% of the total variance. The loading results of each index and factor were good, and the factor structure was consistent with the results of exploratory research. The results of the exploratory factor analysis are shown in Table 7.

#### 6.2.2. Reliability Test

In order to test the reliability of the scale, this study conducted a reliability analysis of the single dimension and the overall scale. The results show that the overall Cronbach’s coefficient of the scale reaches 0.943, and Cronbach’s coefficient of a single dimension is between 0.829 and 0.907, all greater than 0.8. At the same time, Cronbach’s coefficient decreases after deleting a certain item in the dimension. The results show that the scale has high reliability and reliability.

#### 6.2.3. Confirmatory Factor Analysis

##### Model Fit Test

Through confirmatory factor analysis on the four dimensions of involution, this paper verifies the discriminatory validity of inefficient busyness, exhaustion of innovation, promotion anxiety, and internal competition. The results are shown in Table 8. The fitting index $\chi^{2}$/df is acceptable between 2.0 and 5.0, and the smaller the value, the better the model. The approximate error index RMSEA is lower than 0.08, and the goodness of fit index GFI, relative fitting index CFI, and TLI are higher than 0.9, indicating good fitting. From the data in the table, it can be seen that the single-factor model does not distinguish between dimensions, while the two-factor model combines the three dimensions of inefficient busyness, exhaustion of innovation, and promotion anxiety. The three-factor model combines inefficient busyness and exhaustion of innovation. Their acceptance is not ideal.

##### Scale Validity Test

In this study, the structural validity of the scale is tested by calculating and observing the factor load of each item of the scale, and the convergence validity of the scale is tested by the combination of reliability (CR) and the average variance extraction (AVE) of each factor, and the discrimination validity of the scale is tested by judging whether the square root of the average variance extraction (AVE) of each factor is greater than the correlation coefficient between the factor and other factors. It can be seen from Table 9 that the combined reliability CR values of the four factors are all greater than 0.6; The average variance extraction (AVE) was greater than 0.36; The correlation coefficient r between the factors is between 0.617 and 0.691. The arithmetic square root of the factor average variance extraction AVE is greater than the correlation coefficient between the factor and other factors. The results show that the scale has high convergence validity and discrimination validity.

Finally, we determined that the employee involution was a 13-item self-assessment scale with a Likert rating of 5, which was used to evaluate the subjective feelings of employees in involution. The scale is composed of a group of statements. Each statement has five answers: “very agree”, “agree”, “not necessarily”, “disagree”, and “very disagree”, which are respectively recorded as 5, 4, 3, 2, and 1. When the respondents answer the items of this questionnaire, they specifically refer to their own degree of recognition of the statement. The total score of each respondent’s attitude is the sum of his answers to each question, which can explain his attitude or her different states on this scale. The higher the score of the subjects, the higher the degree of involution.

## 7. Discussion

This study introduces the term “involution,” which has been popular in China in recent years, into the field of organizational scholarship and conducts systematic empirical research on employee involution in the workplace. From a macro perspective, the phenomenon of involution is a group crisis and a major challenge in the development of the group work model and management system, whereas, from a micro perspective, the occurrence of involution makes each individual tired of work and affects subjective well-being. Therefore, to solve employee involution, both external and internal approaches, like external and internal adjustment, should be used. This will help employees eliminate the “involution” crisis, get over job burnout, and improve their work enthusiasm and performance. The idea behind this goal is to look into the structural side of employee involution and study how different levels of involution can be measured.

This paper’s research method is grounded theory, and it collects data through in-depth interviews with employees at their workplaces and other means. Through the step-by-step analysis of the performance of employee involution, this paper puts forward the definition of employee involution and extends the connotation of involution based on previous studies. Through constant coding and analysis, this study explores the performance of employee involution in behavior, cognition, emotion, and motivation. Finally, it is concluded that the involution of employees in the workplace contains four dimensions: inefficient busyness, exhaustion of innovation, promotion anxiety, and internal competition, which is a multi-dimensional concept with rich connotations. Secondly, this paper developed and tested the employee involution measurement scale. The research concluded that the employee involution measurement scale includes 4 factors, which are composed of 13 measurement items. The data analysis results show that the reliability and validity are at the ideal level, and it is an effective evaluation scale. Our findings have important implications for both theory and management practice.

### 7.1. Theoretical Implications

First, the article focuses on the phenomenon of involution, which is widespread but lacks systematic research in the academic community. Currently, most academic research on involution is limited to the political, economic, and cultural fields, but few studies on the phenomenon of involution in the workplace are conducted at the micro level. And in recent years, most of the studies have discussed the causes of involution from a single perspective, lacking a theoretical basis and empirical or real-world research. For the organizational phenomenon that is constantly developing and changing, it is not only necessary to confirm it from the perspective of rationality and data, but also to understand the real needs of contemporary employees and the current situation of the organization from the perspectives of “people”, “management”, “system” and “emotions” in the organization. Based on theoretical research, this paper collects employee and network big data in the organization through multiple channels, conducts empirical research on the phenomenon of involution in the workplace from the perspective of employees, and analyzes its mechanism. This is a new exploration of involution in the workplace. This paper defines the connotation of employee involution and analyzes the four dimensions it contains. It is an in-depth study of involution in terms of concept definition, method selection, and research content, which further enriches and expands the theoretical research of involution.

Secondly, the framework established in this study tells decision-makers about the important factors in the formation of employee involution and why. Our research emphasizes that involution is not formed in short-term or single stress, burnout, or anxiety: employee involution is long-term, continuous, and diversified. This research shows the continuous interaction between the organization and individual employees in the process of involution and reveals how the four dimensions of employee involution are reflected in behavior, cognition, emotion, and motivation, as well as the attitude and behavior deviation of employees under the effect of involution, which can inspire enterprise managers to think and adjust the thinking of enterprises to cope with involution from the perspective of employee psychology and individual differences. It provides a reliable theoretical reference for enterprise leaders to effectively understand and deal with employee involution problems.

Finally, turning the research on involution from the political, economic, and cultural fields to the workplace field at the micro level has direct value for the research on involution in the workplace and also has a reference and inspiration for the research on involution in other fields. At the same time, on the basis of rooted exploration, we have compiled a 13-item employee involution measurement scale through in-depth interviews and expert consultation and proved its reliability and validity through empirical research. The scale can be directly applied to quantitative measures related to employee involution topics. The study also has reference value for involution measurement in other populations and contexts. This study provides an important theoretical perspective and research ideas for scholars who study the psychology and behavior of employees in the workplace from the organizational perspective (such as strategic enterprise management and strategic human resources management) and the personal perspective (such as organizational behavior, organizational psychology, and occupational health research).

### 7.2. Management Implications

The relevant research results of this paper provide some practical enlightenment for human resources practitioners and managers to solve employee involution and the resulting adverse effects, which are shown as follows:

(1) To a certain extent, this study has promoted the recognition and attention of various organizations at all levels to involution. Today, the phenomenon of involution in the workplace is becoming more and more obvious. Both employees, companies, and the whole society should be brave to face it, make positive progress, and break the impact of involution. As far as employees are concerned, if they want to really get rid of workplace involution, they must first set practical goals, recognize the core values they provide in their work, and clearly find their workplace anchor and direction, instead of “being involved” with the waves. At the same time, it is necessary to break through the “longboard” of development and improve their competitiveness. After a short period of rapid growth and rise, employees easily entered the platform stage. In this state, it is easy to fall into the dilemma of not advancing or falling back. Although there may be some slight progress after the endless “superficial efforts” work, in the long run, it will inevitably stagnate. Employees seem busy at work but do not have enough nourishment to support people to continue to grow in exploration. They can only stay in a simple self-repeating state for a long time [13] and then enter the trap of “involution”. In addition, some self-regulation strategies, such as strengths use, playful work design, and proactive vitality management [52], will also be effective in dealing with occupational involution. Strengths use means that one can often achieve success by using one’s own advantages, like creativity, while playful work design means redesigning the work experience to make it more interesting and meaningful [53]. Proactive vitality management refers to actively managing one’s own spirit and physical strength so as to be able to effectively handle the next work task [54]. Therefore, employees should take the initiative to challenge the state of their downward development in their individual development, tap their advantages, cultivate their uniqueness, and give play to their autonomy, such as continuously improving their optimal ability in career development, enhancing their core competitiveness, and finding suitable channels for their upward mobility.
(2) For enterprise managers, by breaking the bottleneck of employee involution, the development space of organizations and individuals will be expanded unprecedentedly, and thousands of new innovations will emerge. In specific practice, organizational management should be more personalized, and it is necessary to deeply understand employees’ individual characteristics and high-level psychological needs. Caring about employees’ personal well-being is an indicator of good leadership [55]. Once employees think that the organization treats them well, they will be willing to show behaviors that are beneficial to the organization and reflect them in their work attitude or behavior [56]. Therefore, organizational leaders should care about, recognize, and support employees’ pursuit of work interests and personal values to meet their self-realization needs. Lantara [57] believes that correct work motivation and appreciation of work will lead to higher work enthusiasm. When leaders respect employees’ contributions to the organization and their reasonable opinions and suggestions for the organization, they can effectively correct employees’ work motivation, fully mobilize their work enthusiasm and stimulate their potential. Secondly, managers should provide clear work objectives for employees and improve their sense of self-worth. The goal is one of the sources of motivation. Whether it is a long-term or short-term goal, it can provide employees with work motivation. Hackman and Okiham [58], the proponents of the work characteristic model, believe that intrinsic motivation reflects the efficient working state of employees through self-motivation. If employees are driven by intrinsic motivation, they will feel positive emotional experiences in the work process. Contemporary employees are very concerned about the realization of self-worth. The organization should formulate the responsibilities of each post, clearly divide the work tasks, and let employees understand their work objectives and the effects to be achieved. This way, employees can achieve their objectives while better realizing organizational objectives. Finally, managers must focus on creating a good organizational atmosphere [59], which can improve employees’ performance in the workplace. If the environment does not support it, employees will not have the motivation to do something well. This concept is consistent with the social exchange theory, which is based on the assumption that social exchange involves multiple behaviors that generate obligations and that relationships will increase over time, thus becoming trustworthy, loyal, and mutually committed [60]. The organization should focus on changing the work environment’s characteristics, improving colleagues’ social support [61], and cooperative relations [57]. One of the important characteristics of employee involution is their distrust of interpersonal relationships under high competitive pressure. Greenhalgh and Rosenblatt [62] pointed out that the sense of team psychological security refers to the subjective perception of employees on the objective environment of the work team, not only the working conditions themselves, but also a judgment that individuals can express their personal beliefs, and that “there is no need to worry about the negative consequences on self-impression, identity or occupation” [63], which is also a shared belief of the team that interpersonal risks are safe. It is significant for the team’s execution process, such as team cooperation, communication, shared decision-making, creativity, and team performance [64,65].
(3) Organizations must appropriately develop policies to guide employees’ work values. Organizations should establish correct and positive values in management work, guide employees’ work values to change in a positive and healthy direction, change employees’ work attitudes, and drive employees’ happiness and performance improvement. When employees experience involution, they exhibit coping avoidance and self-sabotage, leading to more anxiety and stress at work. And employees who experience stress show impaired recovery and reduced job shaping, resulting in a lack of personal and job resources and a lack of challenge over time. This gradual stress process will eventually lead to lasting burnout [66], which is not conducive to the output of enterprise innovation and performance. Therefore, the organization can reduce and prevent employee involution by providing stable resources in the form of human resource practice and healthy leadership. Through post-value analysis and enterprise strategic planning, we can establish a performance management system that matches “people, posts, and affairs” driven by post-value reflection, match employees’ income with value contribution, integrate employees’ learning and growth opportunities with performance evaluation results and integrate employees’ advantage orientation and potential development so that each employee can find the most suitable position on the stage and play the perfect role. Through innovative development and the conversion of old and new kinetic energy, we will break through the “involution” development bottleneck of employees and continue improving enterprises’ core competitiveness.

## 8. Conclusions

In order to make up for the deficiency of the research on employee involution at this stage and promote the research progress of employee involution in the field of organization and management on this basis, this study proposed the research question of “What are the contents of employee involution in the workplace”. In this paper, quantitative and qualitative research methods are used to explore the connotation and structural dimensions of employee involution, and on this basis, the measurement scale of employee involution is developed and tested. This research has increased our knowledge and understanding of employee involution. Instead of replacing the current conceptualization of involution, we have determined the conceptual boundary and expanded the current theoretical framework, which is of great and unique significance to the research and development of involution and the practice of organization and management. In addition, in terms of research level, due to the focus on the structural dimension of involution of employee level exploration, there is no further exploration of its specific performance at the organizational level. We hope that future research may further apply more comprehensive data and reasonable theories to explore the structural dimension of involution from more levels.

## Figures and Tables

**Table 1:** Definition of “Involution” in Western academic circles.

Researcher	Definition	Field
Immanuel Kant, 1790	Things don’t reach their advanced or perfect state in the process of development, but they are constantly copied and complicated on the basis of a certain state.	Philosophy
Alexander Goldenweiser, 1936	A cultural phenomenon that is constantly refined and complicated inside.	Anthropology
Clifford Geertz, 1963	The phenomenon that a social or cultural model will stagnate or cannot be transformed into another advanced model after reaching a certain form at a certain stage of development.	Economics
Phillip C. C. Huang, 1986	The marginal return of labor force decreases.	Political science
Prasenjit Duara, 1988	(A state institution) Reproducing or expanding an old state or social system to expand its administrative functions.	Political science

**Table 2:** Statistical information of respondents.

Attributes		Number	Proportion (%)
Interview method	Face-to-face interview	26	25.24
	Network Interview	77	74.76
Gender	Male	58	56.31
	Female	45	43.69
Occupation	Education, scientific research, professional and technical personnel	37	35.92
	Enterprises and institutions, managers	42	40.78
	Commerce, service industry, and others	24	23.30
Age	20–30 years old	55	53.40
	31–45 years old	30	29.13
	Over 45 years old	18	17.47

**Table 3:** Process and Results of Open Coding (Excerpt).

Original Data Statement (Representative Statement)	Category
R10 The current job is relatively exhausting, because after working in this industry for many years, as a woman, the work pressure in this industry is also very high.	Internal pressure
R08 Because everyone around me is working hard, and I also want to be a better teacher, so I force myself to do some work to improve myself.	Forced competition
R24 They will also cope with the work with the idea that more things are worse than less things, and everyone is also seeking advantages and avoiding disadvantages.	Passive execution
R27 Every day I pick up people in the same city, doing the same things and looking at the same things.	Repeating work
R05 Tired, there are many account books to read every day, and my physical strength can’t keep up.	Excessive consumption
R05 It feels so powerless, but the fact is that it is difficult to break through.	Work inefficiency
R08 Because our own teaching methods have formed a relatively fixed pattern, it is difficult to change.	Path dependency
R25 For problems that might be explained by simple regression or correlation analysis, it is necessary to set a complex model to be a good article. Otherwise, there is insufficient innovation and depth.	Simplify to complex
R26 Now I feel that I can develop well, but I have no ability to improve. Now I can live a stable life, even if I do the same job every day, but as long as the job is stable.	Self-locking
R46 Work really consumes most of my energy, I feel that I can’t take care of many other things, and I don’t want to think about it.	Difficulty concentrating
R05 I have been confused and don’t know what to do. I have taken many certificates, but I can’t use them.	Uncertain future
R35 When something goes wrong at work, you are in a bad mood. When talking with family and friends, you may accidentally bring negative emotions into your life.	Unregulated emotions
R06 Doing repetitive work every day will make you feel boring, and you will lose a lot of fun in your life.	Work boring

**Table 4:** Process and results of axial coding.

Main Category	Corresponding Category	Category Connotation
Inefficient busyness	Repeating work	Do the same work, the same method, and the same amount of work every day.
	Excessive consumption	Work overtime and consume a lot of energy.
	Work inefficiency	The work efficiency is low, there is no breakthrough, and it has been spinning in place.
Exhaustion of innovation	Path dependency	Due to the limitations of cognition, when individuals face complex decisions, their thinking patterns rely on past scenarios to interpret and respond.
	Simplify to complex	The work pursues refinement, but it is meaningless to work improvement. It just complicates simple problems.
	Self-locking	Have higher pursuits but can only be content with the status quo because of self-doubt.
	Difficulty concentrating	Work is so exhausting that you can’t concentrate and think about problems after work is over.
Promotion anxiety	Uncertain future	Anxiety about the future and progress, unclear future direction.
	Work boring	The work content is monotonous and boring.
	Unregulated emotions	Bring work emotions to life.
Internal competition	Internal pressure	Competitive pressure from leadership and within the industry.
	Passive execution	In order to avoid responsibility, passively cope with leadership work.
	Forced competition	Force yourself to work hard out of anxiety about the promotion of others.

**Table 5:** Results of selective coding.

Core Category	Typical Relationship Structure	Connotation of Relationship Structure
Involution	Inefficient busyness →Involution	Inefficiency busyness is the behavior of employees who are in a state of involution, and it is an important part of the manifestation of involution.
	Exhaustion of innovation →Involution	Exhaustion of innovation refers to the lack of employees’ awareness of innovation and the limited thinking mode, which is the cognitive performance of employees’ involution state.
	Promotion anxiety →Involution	Promotion anxiety expresses the anxiety, boredom, and irritability of employees, which is reflected in the emotional dimension of involution.
	Internal competition →Involution	Internal competition is embodied in three aspects: internal pressure, passive execution, and forced competition, which is the embodiment of involution in the dimension of motivation.

**Table 6:** Demographic data.

Statistical items	Category	Frequency	Percentage
Gender	Male	205	46.6
	Female	235	53.4
Age	Under 30	170	38.6
	31–40 years old	143	32.5
	41–50 years old	84	19.1
	Over 50	43	9.8
Education	Bachelor’s degree or above	148	33.6
	Junior college	102	23.2
	High school and technical secondary school	103	23.4
	Junior high school and below	87	19.8
Position	Ordinary workers	154	35
	Junior managers	104	23.6
	Middle management	122	27.7
	Senior management	60	13.6

**Table 7:** Exploratory factor analysis results of employee involution scale.

Measurement Item	1	2	3	4
I do repetitive work every day	0.789			
It’s hard for me to make a new breakthrough in my work	0.779			
I often work overtime doing meaningless work	0.686			
My work is very tedious, so it will consume a lot of energy	0.662			
I don’t know how to change my job situation		0.781		
I don’t know how to pursue a better job		0.774		
I didn’t have time to think of more efficient ways to work		0.768		
I always deal with work problems in a fixed way		0.753		
I often complicate simple problems		0.751		
I’m worried about my job prospects			0.817	
I often feel irritable because of my work			0.808	
I often feel anxious and restless			0.793	
I think my job is boring			0.763	
I’m worried that my job performance will be lower than others				0.783
My leaders and colleagues always put a lot of pressure on me				0.779
There is a lot of internal competition pressure in my industry				0.763
Seeing others working hard, I will force myself to work				0.735
I often deal with leadership work passively				0.712

**Table 8:** Four-dimensional confirmatory result analysis of involution.

Model	$\chi^{2}$	DF	$\chi^{2}/\mathrm{df}$	RMSEA	CFI	TLI	IFI
Original model	328.912	129	2.550	0.059	0.971	0.965	0.971
Three-factor model	696.377	132	5.276	0.099	0.917	0.904	0.918
Two-factor model	1308.920	134	9.768	0.141	0.828	0.804	0.828
One factor model	1754.579	135	12.997	0.165	0.763	0.731	0.764

**Table 9:** The correlation coefficient, CR value, and AVE value of each factor.

	1	2	3	4
Inefficient busyness	(0.724)			
Exhaustion of innovation	0.691 **	(0.747)		
Promotion anxiety	0.654 **	0.617 **	(0.792)	
Internal competition	0.689 **	0.697 **	0.677 **	(0.736)
AVE	0.535	0.586	0.633	0.570
CR	0.820	0.876	0.873	0.869

Note: ** means *p* < 0.01, and the square root of the factor average variance extraction AVE is in the diagonal brackets.

## Data Availability

Publicly available datasets were analyzed in this study. This data can be found here: https://kdocs.cn/l/ctdgCLtJfNBF.

## References

[1] Kim S., Kwon K., Wang J. (2020). Impacts of job control on overtime and stress: Cases in the United States and South Korea. Int. J. Hum. Resour. Manag..

[2] Feldman D.C. (2002). Managers’ propensity to work longer hours. Hum. Resour. Manag. Rev..

[3] Zhao M., Beveridge A.J. (2022). Neijuan: An Investigation of Overtime Work Among Chinese Urban White-Collar Employees. Acad. Manag. Proc..

[4] Meng S., Wei B., Xu G., Zhang R. Analysis of Enterprise Operation Under the Impact of COVID-19 Epidemic: A Case Study of Nike Inc. Proceedings of the 2021 International Conference on Financial Management and Economic Transition (FMET 2021).

[5] Li R. Analysis on the Chinese Anxiety of Involution from *Jiwa* with the Background of Globalization. Advances in Social Science, Education and Humanities Research, Proceedings of the 2021 4th International Conference on Humanities Education and Social Sciences (ICHESS 2021), Xishuangbanna, China, 29–31 October 2021.

[6] Rottinghaus P.J., Jenkins N., Jantzer A.M. (2009). Relation of Depression and Affectivity to Career Decision Status and Self-Efficacy in College Students. J. Career Assess..

[7] Chisholm D., Sweeny K., Sheehan P., Rasmussen B., Smit F., Cuijpers P., Saxena S. (2016). Scaling-up treatment of depression and anxiety: A global return on investment analysis. Lancet Psychiatry.

[8] George G., Howard-Grenville J., Joshi A., Tihanyi L. (2016). Understanding and tackling societal grand challenges through management research. Acad. Manag. J..

[9] Mulvey B., Wright E. (2022). Global and local possible selves: Differentiated strategies for positional competition among Chinese university students. Br. Educ. Res. J..

[10] Qianni W., Shifan G. (2020). How One Obscure Word Captures Urban China’s Unhappiness: Anthropologist Xiang Biao Explains Why the Academic Concept of “Involution” Became a Social Media Buzzword. Sixth Tone.

[11] Liu B., Chen H., Hou C., Wang Y. (2021). The structure and measurement of overtime work: A scale development study among Chinese employees. Curr. Psychol..

[12] Bao X. (2022). The Striving Trap: Chinese 996 Work Culture, Online and Offline Perspectives. Doctoral Dissertation.

[13] Li C. From Involution to Education: A Glance to Chinese Young Generation. Advances in Social Science, Proceedings of the 2021 4th International Conference on Humanities Education and Social Sciences (ICHESS 2021), Xishuangbanna, China, 29–31 October 2021.

[14] Van der Hulst M., Geurts S. (2001). Associations between overtime and psychological health in high and low reward jobs. Work Stress.

[15] Beckers D.G., Van der Linden D., Smulders P.G., Kompier M.A., Taris T., Geurts S.A. (2008). Voluntary or involuntary? Control over overtime and rewards for overtime in relation to fatigue and work satisfaction. Work Stress.

[16] Dettmers J. (2017). How extended work availability affects well-being: The mediating roles of psychological detachment and work-family-conflict. Work Stress.

[17] Cheng B.H., McCarthy J.M. (2018). Understanding the dark and bright sides of anxiety: A theory of workplace anxiety. J. Appl. Psychol..

[18] Eysenck M.W., Derakhshan N., Santos R., Calvo M. (2007). Anxiety and cognitive performance: Attentional control theory. Emotion.

[19] Theorell T., Hammarström A., Aronsson G., Bendz L.T., Grape T., Hogstedt C., Marteinsdottir I., Skoog I., Hall C. (2015). A systematic review including meta-analysis of work environment and depressive symptoms. BMC Public Health.

[20] Stansfeld S., Candy B. (2006). Psychosocial work environment and mental health—A meta-analytic review. Scand. J. Work Environ. Health.

[21] Nieuwenhuijsen K., Bruinvels D., Frings-Dresen M. (2010). Psychosocial work environment and stress-related disorders, a systematic review. Occup. Med..

[22] Fila M.J., Purl J., Griffeth R.W. (2017). Job demands, control and support: Meta-analyzing moderator effects of gender, nationality, and occupation. Hum. Resour. Manag. Rev..

[23] Siegrist J. (1996). Adverse health effects of high-effort/low-reward conditions. J. Occup. Health Psychol..

[24] Naidoo S., Sutherland M. (2016). A management dilemma: Positioning employees for internal competition versus internal collaboration. Is coopetition possible?. S. Afr. J. Bus. Manag..

[25] Tjosvold D., Johnson D.W., Johnson R.T., Sun H. (2006). Competitive motives and strategies: Understanding constructive competition. Group Dyn. Theory Res. Pract..

[26] Khoja F. (2008). Is sibling rivalry good or bad for high technology organizations?. J. High Technol. Manag. Res..

[27] Yari M., Monjezi M., Bagherpour R., Sayadi A.R. (2014). Blasting Operation Management Using Mathematical Methods. Engineering Geology for Society and Territory-Volume 1.

[28] Yari M., Bagherpour R., Khoshouei M., Pedram H. (2020). Investigating a comprehensive model for evaluating occupational and environmental risks of dimensional stone mining. Rud. Zb..

[29] Meng-ying L. (2021). “Nei Juan” in Exam-oriented Education in China. J. Lit. Art Stud..

[30] Wei S. (2006). Smith Power and Brodale Bell Jar—A possible new perspective to study the historical reasons for the rise of the western world in modern times and the relative stagnation of the late Qing empire. Soc. Sci. Front..

[31] Geertz C. (1963). Agricultural Involution: The Process of Ecological Change in Indonesia/Clifford Geertz.

[32] Yao X. (1992). China’s rural areas are getting rid of the poverty trap of “growth without development”. Acad. Q. Shanghai Acad. Soc. Sci..

[33] Du Z. (2000). Culture, Power and Nation State—Interview with Professor Du Zanqi. Xuehai.

[34] Liu S., Qiu Z. (2004). Analysis of the concept of “involution”. Sociol. Res..

[35] Liu Y. (2021). China’s “Involuted” Generation: A New Word Has Entered the Popular Lexicon to Describe Feelings of Burnout, Ennui, and Despair. https://productivityhub.org/2021/05/15/chinas-involuted-generation/.

[36] Wang F., Wang Y. (2021). The Buzzwords Reflecting the Frustration of China’s Young Generation.

[37] Qin G. (2021). China in Hot Words—Ambassador Qin Gang’s Keynote Speech at the Theme Forum of “Tourism and People to People Exchange” of the American Asian Society. https://new.qq.com/rain/a/20211008A01L3O00.

[38] Steel Z., Marnane C., Iranpour C., Chey T., Jackson J.W., Patel V., Silove D. (2014). The global prevalence of common mental disorders: A systematic review and meta-analysis 1980–2013. Int. J. Epidemiol..

[39] Barsky A.P., Kaplan S.A. (2007). If you feel bad, it’s unfair: A quantitative synthesis of affect and organizational justice perceptions. J. Appl. Psychol..

[40] Amabile T.M., Barsade S.G., Mueller J.S., Staw B.M. (2005). Affect and Creativity at Work. Adm. Sci. Q..

[41] Kim P.B., Murrmann S.K., Lee G. (2009). Moderating effects of gender and organizational level between role stress and job satisfaction among hotel employees. Int. J. Hosp. Manag..

[42] Ng T.W.H., Feldman D.C. (2011). Employee voice behavior: A meta-analytic test of the conservation of resources framework. J. Organ. Behav..

[43] Schaufeli W.B., Leiter M.P., Maslach C. (2010). Burnout: 35 Years of research and practice. IEEE Eng. Manag. Rev..

[44] Fisherman S. (2015). Emotional Well-Being as a Function of Professional Identity and Burnout among Homeroom and Subject Teachers. Res. J. Educ..

[45] Bianchi R., Schonfeld I.S., Laurent E. (2017). Burnout or depression: Both individual and social issue. Lancet.

[46] Glaser B.G., Strauss A.L., Strutzel E. (1967). The discovery of grounded theory: Strategies for qualitative research. Nurs. Res..

[47] Glaser B.G., Strauss A. (2017). The Discovery of Grounded Theory: Strategies for Qualitative Research.

[48] Corbin J., Strauss A. (2008). Basic of Qualitative Research, (3rd ed.): Techniques and Procedures for Developing Grounded Theory.

[49] Dey I. (2004). Grounded theory. Qual. Res. Pract..

[50] Charmaz K. (2000). Grounded theory: Objectivist and constructivist methods. Handb. Qual. Res..

[51] Fassinger R.E. (2005). Paradigms, praxis, problems, and promise: Grounded theory in counseling psychology research. J. Couns. Psychol..

[52] van Wingerden J., Bakker A.B., Derks D. (2016). The longitudinal impact of a job crafting intervention. Eur. J. Work Organ. Psychol..

[53] Scharp Y.S., Breevaart K., Bakker A.B., van der Linden D. (2019). Daily playful work design: A trait activation perspective. J. Res. Personal..

[54] Kamp E.M.O.D., Tims M., Bakker A.B., Demerouti E. (2018). Proactive vitality management in the work context: Development and validation of a new instrument. Eur. J. Work Organ. Psychol..

[55] Hon A.H. (2013). Does job creativity requirement improve service performance? A multilevel analysis of work stress and service environment. Int. J. Hosp. Manag..

[56] Shore L.M., Tetrick L.E. (1991). A Construct Validity Study of the Survey of Perceived Organizational Support. J. Appl. Psychol..

[57] Lantara A.N.F. (2019). The effect of the organizational communication climate and work enthusiasm on employee performance. Manag. Sci. Lett..

[58] Hackman J.R., Oldham G.R. (1975). Development of the Job Diagnostic Survey. J. Appl. Psychol..

[59] Arakal T., Mampilly D. (2013). The Impact of Organizational Climate on Performance of Employees, Trends and Challenges in Global Business Management. http://www.conference.bonfring.org/papers/sngce_placitum2013/hrm04.pdf.

[60] Cropanzano R., Mitchell M.S. (2005). Social Exchange Theory: An Interdisciplinary Review. J. Manag..

[61] Heaney C.A., Israel B.A., Schurman S.J., Baker E.A., House J.S., Hugentobler M. (1993). Industrial relations, worksite stress reduction, and employee well-being: A participatory action research investigation. J. Organ. Behav..

[62] Greenhalgh L., Rosenblatt Z. (1984). Job Insecurity: Toward Conceptual Clarity. Acad. Manag. Rev..

[63] Kahn W.A. (1990). Psychological Conditions of Personal Engagement and Disengagement at Work. Acad. Manag. J..

[64] Bradley B.H., Postlethwaite B.E., Klotz A.C., Hamdani M.R., Brown K.G. (2012). Reaping the benefits of task conflict in teams: The critical role of team psychological safety climate. J. Appl. Psychol..

[65] Tynan R. (2005). The Effects of Threat Sensitivity and Face Giving on Dyadic Psychological Safety and Upward Communication1. J. Appl. Soc. Psychol..

[66] Bakker A.B., de Vries J.D. (2020). Job Demands—Resources theory and self-regulation: New explanations and remedies for job burnout. Anxiety Stress Coping.

